# Customization of neonatal functional magnetic resonance imaging: A preclinical phantom-based study

**DOI:** 10.1371/journal.pone.0313192

**Published:** 2024-11-01

**Authors:** Juan F. Quinones, Tina Schmitt, Tommaso Pavan, Andrea Hildebrandt, Axel Heep

**Affiliations:** 1 Psychological Methods and Statistics, Department of Psychology, School of Medicine and Health Sciences, Carl von Ossietzky Universität Oldenburg, Oldenburg, Germany; 2 Cluster of Excellence Hearing4all, Carl von Ossietzky Universität Oldenburg, Oldenburg, Germany; 3 Neuroimaging Unit, School of Medicine and Health Sciences, Carl von Ossietzky Universität Oldenburg, Oldenburg, Germany; 4 Department of Radiology, Lausanne University Hospital (CHUV), Lausanne, Switzerland; 5 School of Biology and Medicine, University of Lausanne, Lausanne, Switzerland; 6 Research Center Neurosensory Science, Carl von Ossietzky Universität Oldenburg, Oldenburg, Germany; 7 Perinatal Neurobiology Group, Department of Pediatrics, School of Medicine and Health Sciences, Carl von Ossietzky Universität Oldenburg, Oldenburg, Germany; Pisa University Hospital, ITALY

## Abstract

Over the past few decades, the use of functional magnetic resonance imaging (fMRI) on neonates and very young children has increased dramatically in research and clinical settings. However, the specific characteristics of this population and the MRI standards largely derived from adult studies, pose serious practical challenges. The current study aims to provide general methodological guidelines for customized neonatal fMRI by assessing the performance of various fMRI hardware and software applications. Specifically, this article focuses on MR equipment (head coils) and MR sequences (singleband vs. multiband). We computed and compared the signal-to-noise ratio (SNR) and the temporal SNR (tSNR) in different fMRI protocols using a small-size spherical phantom in three different commercial receiver-only head-neck coils. Our findings highlight the importance of coil selection and fMRI sequence planning in optimizing neonatal fMRI. For SNR, the prescan normalize filter resulted in significantly higher values overall, while in general there was no difference between the different sequences. In terms of head coil performance, the 20-channel head coil showed slightly but significantly higher values compared to the others. For tSNR, there was no difference in the usage of the prescan normalize filter, but the values were significantly higher in the singleband EPI sequences compared to the multiband. In contrast to the SNR, the pediatric head coil seems to have an advantage for tSNR. We provide five practical guidelines to assist researchers and clinicians in developing fMRI studies in neonates and young infants. These recommendations are especially relevant considering ethical constraints and exogenous challenges of neonatal fMRI.

## Introduction

Magnetic resonance imaging (MRI) of newborns has proven essential in understanding brain functioning and brain development at cellular [1], microstructural [2–4] and connectivity [5] levels. Functional MRI (fMRI) can be used in clinical settings to non-invasively study brain development and brain injury, and in research settings to characterize metabolic processes [6], elucidate neonatal brain networks [7–9] and describe neural correlates of sensation and motion [10–13]. Despite the growing interest in neonatal fMRI, reflected in large-scale neuroimaging projects, for instance the Developing Human Connectome Project [dHCP; 14], its usage faces formidable challenges [15–21].

Intrinsic challenges of fMRI in neonates relate to tissue properties that are typical at very young ages and drastically different from those observed in adults. The most noticeable differences are the overall brain size and microstructural properties [22, 23], which vary across the brain and change over very short time frames (e.g., dendritic arborization and axonal packing). Further, water and fat content in the newborn brain result in different T1 and T2 relaxation times as compared to adults [1, 16, 24]. This affects signal intensities and questions the suitability of standard (adult) fMRI acquisition parameters. Other intrinsic challenges relate to safety and the high demands of collecting good quality data. In-scanner monitoring and motion artefact prevention are crucial in clinical and research settings. In most cases, a dedicated medical team will constantly monitor the newborn’s vital signs to rule out any potential risks [18, 20]. In research settings, it is unusual to sedate neonates for scanning, but natural sleep is induced instead. However, this is no guarantee of movement-free measurements and scanning sessions often need to be interrupted or even aborted.

Extrinsic challenges generally refer to the ethical concerns raised by testing individuals who are unable to express consent and highly sensitive to environmental stress. While clinical MRI scans are necessary from a medical point of view, neonatal fMRI for research purposes undergoes an exhaustive ethical review. Furthermore, neonatal fMRI researchers typically face methodological issues when planning studies and collecting data. For example, ethical committees are unlikely to approve the scanning of neonates to test and refine protocol parameters to establish data collection pipelines. This entails a high risk of collecting unusable data or data of suboptimal quality.

The field has coped with some of the challenges of neonatal fMRI described above by making modifications to the MRI hardware, the fMRI sequences and the scanning procedure. A remarkable example is the development of customized dedicated neonatal head coils. Overall, a larger filling factor is accompanied by a larger SNR [11, 18, 25, 26]. Other hardware modifications, such as customized incubators and MRI scanners have been developed independently or integrated with customized coils, also able to adapt to complex and variable structures, to enhance the quality of neonatal MRI data [for reviews see 15, 18, 20, 27–29].

Concerning fMRI sequences, protocol parameters have been adjusted to the tissue-dependent T1 and T2 relaxation times for MRI scanners of different magnetic field strength [16, 19, 20]. Characterizing brain activity in the neonatal brain via blood oxygenation level dependent (BOLD) signal fluctuations with resting-state fMRI (rs-fMRI) requires rapid data acquisition, which can be achieved with an accelerated multiband fMRI sequence [30], although the use of acceleration methods can lead to a reduction in SNR [31] and temporal SNR (tSNR; [32]). There are several ways to calculate the SNR [see 32, 33, for an overview, 34], depending on different factors which need to be considered for the calculation of the SNR, such as the influence of multichannel combinations, various reconstruction techniques or parallel imaging [35, 36]. Furthermore, noise can be measured in different regions of the image itself, either as background noise outside the interesting signal or background noise within the signal itself. The background noise not related to the signal itself represents just the noise of the system, whereas the noise within the signal also includes patient related noise, such as physiological noise (cardiac and respiratory pulsation, motion), partial volume effects or flow artifacts [37]. Note that the latter is not relevant for the current study, since we only use phantom data. For fMRI studies, with the goal to detect small fluctuations over multiple measurements, the SNR as a static value might not be sufficient. Thus, the tSNR can be used to calculate the SNR of time series which uses the mean over the time series [34].

The use of prescan normalize filter can improve MRI data quality [33]. One of the disadvantages of coils with a high number of channels is a non-uniformity of the signal, i.e. signals deeper in the brain will be smaller whereas those from the cortex are larger [38], resulting in a bright surface signal. There are different methods to correct for this signal non-uniformities, whereas one possibility is the prescan normalize filter which is implemented in Siemens MRI system starting from software version VE11. This function uses a calibration scan with the body coil to measure a phase reference image which is then used to correct the surface coil image [see 33, for a more detailed description of the origin and calculation of the prescan normalization as well as the advantages and disadvantages].

Finally, modifications to the scanning procedure include preparation, in-scan monitoring and detailed documentation of scanning sessions. For instance, the Baby Connectome Project [39] provides a detailed description of participant preparation before data collection, including training sessions. Similarly, the dHCP has developed MRI sequences and protocols that can be interrupted and resumed if the newborn wakes up while scanning. Monitoring and recording physiological data during fMRI data acquisition allows to apply correction methods during the data preprocessing stages [30], for instance to account for blood flow changes locked to cardiac and respiratory activity.

### The present study

Despite the growing interest in neonatal fMRI and fruitful efforts to overcome its difficulties, setting up fMRI measurements remains extremely challenging: First, dedicated neonatal MRI hardware may not be available at all neuroimaging centers. Second, MRI sequences and protocol parameters are rarely transferable among MRI scanner vendors and models without side effects, limiting the use of publicly available MRI protocols. Third, assuming compatibility between MRI scanners, publicly available descriptions of MRI sequences and protocol parameters are often incomplete or may require specialized knowledge. Fourth, assistance services provided by MRI vendors may not cover neonatal imaging. Lastly, the specific absorption rate calculation is based on adult data and must be recalculated for neonatal data to allow the MRI to run the sequences with the necessary safety parameters.

In order to address some of the above challenges, the main goal of the present study is to evaluate the performance of dedicated hardware and fMRI sequences available in the context of neonatal fMRI. We compared SNR and tSNR measures resulting from a series of functional images acquired with three different head coils and a small spherical phantom mimicking the head size of a newborn. We hypothesized that SNR and tSNR would vary as a function of head coil size, and specific sequences and protocol parameters, with higher SNR and tSNR for smaller head coils and the usage of the prescan normalize filter. Our results will contribute to technological development in the growing literature on neonatal fMRI and will provide a quantitative summary of differences among head coils, sequences and protocol parameters. We expect the current study to assist clinicians and researchers in the early stages of projects involving functional imaging of neonatal brains, especially when protocol optimization cannot be carried out with human neonates.

## Materials and methods

### Hardware

Measurements are performed on a 3T MRI scanner (Siemens Magnetom Prisma, VE11C) with three different commercial receiver-only head-neck coils (Siemens) available at the Neuroimaging Unit of the Carl von Ossietzky Universität Oldenburg. The 20-channel head coil consists of 20 integrated pre-amplifiers arranged on two rings with eight elements each and one ring with four elements. The head coil has a vertical inner diameter of 26.5 cm and a horizontal inner diameter of 23 cm. The 64-channel head coil consists of an anthropomorphic geometry with 64 integrated pre-amplifiers, whereas the upper coil part consists of 24 elements, and the lower coil part of 40 elements. The head coil has a vertical inner diameter of 22 cm and a horizontal inner diameter of 19.5 cm. Both head coils are dedicated for usage in adults. The pediatric head coil (QED provided by Siemens) consists of 16 channels, 13 channels for head and three channels for neck imaging. The coil enables fast, high-resolution examinations and is specifically designed for children up to 18 months. This head coil has a vertical inner diameter of 19.5 cm and a horizontal inner diameter of 16.5 cm. One additional advantage of this head coil is that a cradle is provided to allow a safe and efficient transport and positioning of the child.

For our study we used the spherical Funstar Jr phantom (Gold Standard Phantoms, www.goldstandardphantoms.com) with 11 cm in diameter, 34.5 cm circumference and 700 ml volume filled with a chemically cross-linked form of polyacrylamide. This filling is resulting in a super-absorbent, viscoelastic gel which is an update to the traditional agar fBIRN phantom.

**Fig 1:**
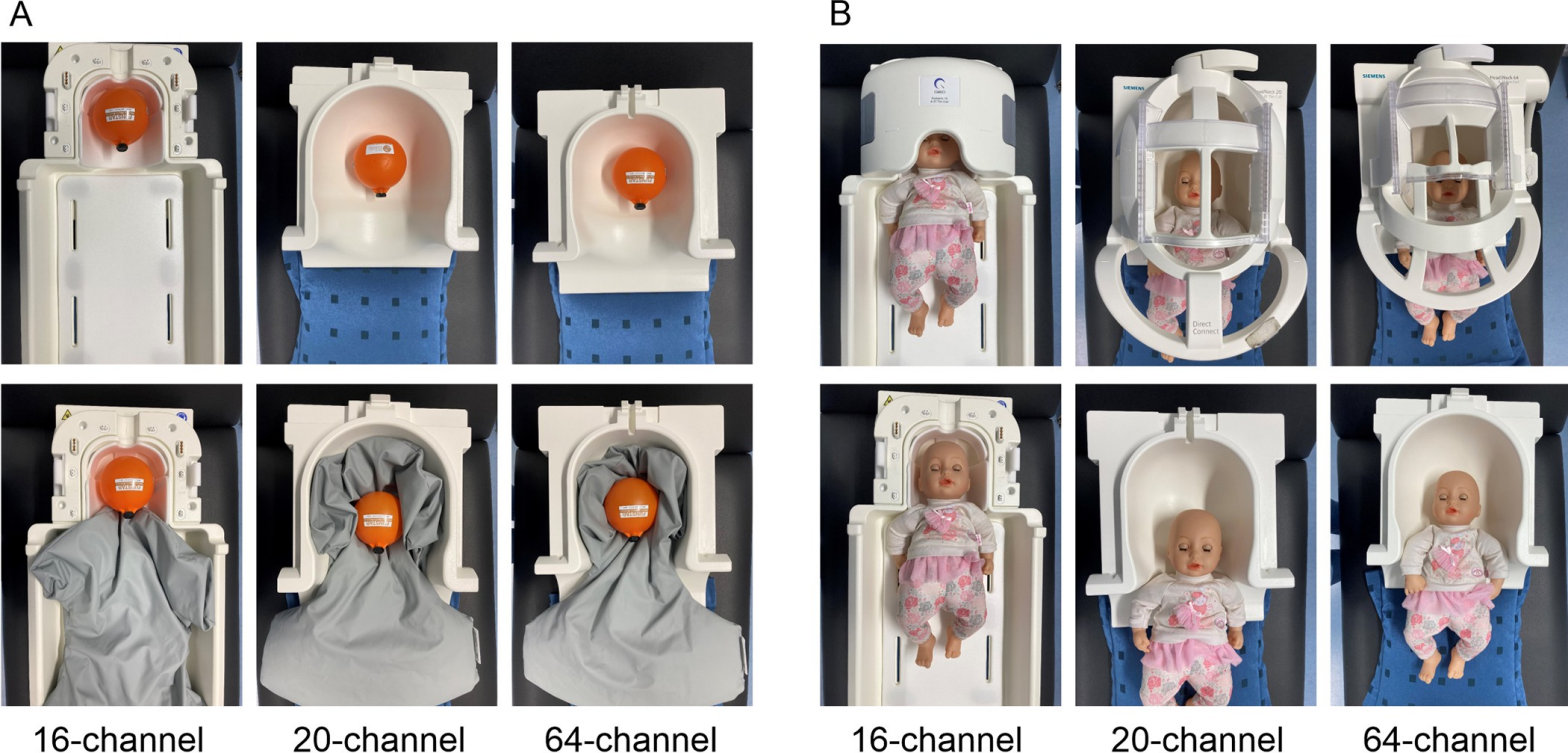
Different head coils used in the study with (A) the Funstar Jr phantom (circumference = 34.5 cm) and (B) a doll representing a newborn (circumference = 34 cm).

Images of the different head coils with the Funstar Jr phantom (A) as well as a doll representing a newborn’s head (head circumference = 34 cm; B) are shown in Fig 1 for visualization purposes of the filling of the head coils. The filling factor of the different head coils can be calculated as the ratio between the phantom and the coil inner volumes (i.e., capacity): phantom ml/coil ml. It ranges between 0 and 1 with higher filling factor values closer to 1. Main geometric properties of the three head coils used in the present study are shown in Table 1.

**Table 1:** Geometric properties of the different head coils.

Head coil	Inner vertical diameter [cm]	Inner horizontal diameter [cm]	Average distance between phantom and coil border [cm]	Capacity [l]	Filling factor
16-channel	19.5	16.5	4.8	2.5	0.28
20-channel	26.5	23	7.9	8.5	0.08
64-channel	22	19.5	6.4	6.5	0.11

### Sequences

Different fMRI sequences for neonatal MRI were compared with variations in some protocol parameters (see Table 2 for more details).

**Table 2:** Overview of the sequences and protocols used with the different head coils.

Name	Slices	Resolution	Voxelsize [mm]	Bandwidth [Hz]	TE [ms]	TR [ms]	Flip Angle	TA [min]
EPI	30	64x64	2.5 x 2.5 x 2.5	1700	30	2000	90	05:04
CMRR1	30	64x64	2.5 x 2.5 x 2.5	1700	30	2030	90	05:09
CMRR3	30	64x64	2.5 x 2.5 x 2.5	1700	30	906	60	02:22
CMRR8	72	104x104	2 x 2 x 2	2290	37	770	52	02:05
MB3	30	64x64	2.5 x 2.5 x 2.5	2790	30	906	60	02:21

A standard EPI sequence was used with the protocol parameters described in [30], where they tested newborns in a 3T Siemens Skyra with the 32-channel head coil (named in the following “EPI”). Smith-Collins et al. (2015) compared the results of the standard EPI with the CMRR multiband sequence [40, 41], which was also used in the current study. To compare the results, we varied the multiband factor between 1 (“CMRR1”) and 3 (“CMRR3”). In addition, we also included the protocol of the dHCP, which was available for the Siemens Prisma (software version VE11C) and the 32-channel head coil, which also used the CMRR multiband sequence (“CMRR8”). However, the 32-channel head coil was not available at our facility. As a comparison for the CMRR multiband sequence also the Siemens multiband sequence was set up with similar parameters as the CMRR sequence used in [30] (“MB3”).

All measurements were performed twice, with and without the prescan normalize filter to reassess effects previously demonstrated by Schmitt and Rieger [33]. In total, the same dataset was measured four times to achieve repeated measures for statistical analyses. GRAPPA and Partial Fourier were OFF during all measurements, since GRAPPA can result in a reduction of SNR due the reduced number of acquired k-space lines [42, 43].

### Data acquisition

The phantom was placed in the respective head coil via the Siemens ComfortKit, a vacuum cushion system to allow a stable positioning of the phantom within the head coil and avoid moving of the phantom due to possible vibrations of the MRI system (see Fig 1). Following each other, the sequences from Table 2 were measured with each of the available head coils. In each of the four runs, 150 scans were acquired to allow better comparability between acquisition times from standard and multiband sequences. Thereafter, the head coil was changed, and the same sequences were run again without any adjustment of the parameters to ensure comparability between the head coils.

### Data analyses

SNR is a measure of the MRI signal relative to the noise in the measurement. SNR increases linearly with field strength and is largely influenced by the features of the receiver coil [29, 33, 34], the geometry and size of the individual being tested, as well as the distance of the measured object from the coil surface [44]. Finally, MRI acquisition parameters also affect the SNR. In particular, shortening the echo time (TE; i.e., time between radio-frequency pulse application and signal collection) is associated with increasing SNR, but also with loss of tissue contrast in T2 images, which are widely used in neonatal MRI.

There are several ways to compute SNR [33, 34], some of which are available to researchers and clinicians as part of quality-check toolboxes, for example the MRIQC tool [45]. However, MRIQC compares individual data to a standard adult brain, which makes the toolbox less useful in neonatal neuroimaging.

In the present study, for each sequence and head coil, SNR was calculated on 4D-averaged phantom data according to the procedure implemented in the MRIQC tool [45], known as a standard tool for MRI quality control.

$$SNR=\frac{I_{f}}{\sigma_{f}\sqrt{n/(n-1)}}$$

where, *I*_*f*_ is the mean across voxel intensities in the image foreground, *ơ* is the standard deviation of the image foreground and *n* is the number of voxel in the foreground. The foreground mask was extracted via FSL 6.0.3 BET. For better visualization purposes, SNR homogeneity maps were calculated with the same SNR equation (eq. 1), with the voxel-wise image intensities as enumerator instead.

The tSNR was calculated by averaging the foreground of the tSNR map of each image. The tSNR maps are computed with the Nipype tSNR implementation (https://doi.org/10.3389/fninf.2011.00013) using the equation:

$$tSNR=\frac{I_{ft}}{\sigma_{ft}}$$

where *I_ft_* corresponds to each foreground voxel’s mean value along the time series, and *σ_ft_* corresponds to each foreground voxel’s standard deviation along the same time series.

For statistical comparisons between sequences, coils and the effect of the prescan normalize filter on SNR and tSNR, we estimated linear mixed effects models (LMM) over the four measurement repetitions. In these models, SNR vs. tSNR was predicted by the repeated measures factors sequence, coil and prescan with levels 5*3*2. In both models, dummy coding was used with CMRR1, 16-channel head coil and prescan normalize off as reference categories for the respective factors.

## Results

### SNR

**Fig 2:**
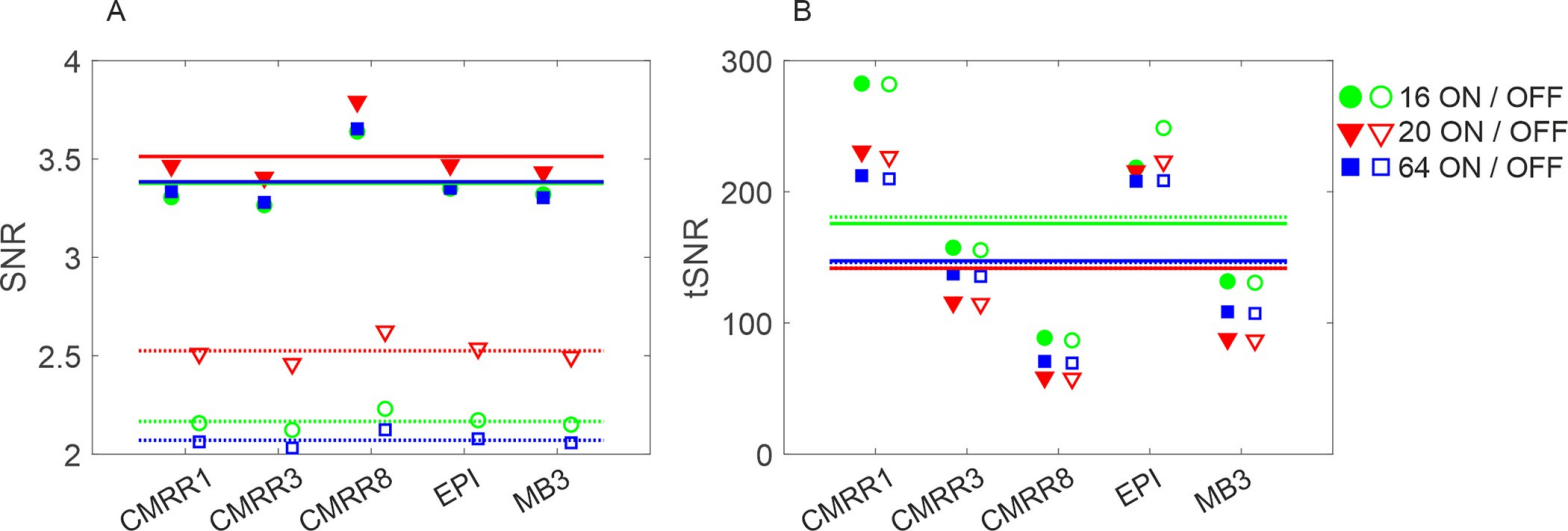
Mean and standard deviation of the SNR (A) and tSNR (B) calculated from the four runs for the different head coils (16-channel in green, 20-channel in red, 64-channel in blue) and prescan normalize filter (ON = filled symbols, with mean shown as a solid line; OFF = not filled symbols, with mean shown as dashed line) for the different sequences. For SNR differences are larger for prescan normalize ON (filled, solid line) compared to OFF (not filled, dashed line) with less differences between the different sequences, whereas tSNR shows no differences for the usage of the prescan normalize filter, but for different sequences (note that tSNR results will be described in the following paragraph).

The SNR in the different measurements showed an overall higher value throughout all sequences (see S1 Table), when the prescan normalize filter was turned ON (Fig 2A, filled, solid line indicates the mean), compared to when the prescan normalize filter was turned OFF (Fig 2A, not filled, dashed line indicates the mean). The model accordingly revealed a significant main effect (parameterized by the regression coefficient *b*) of the factor prescan (*b* = 1.17, *t* = 41.17, *p* < 0.01). Comparisons of the different head coils revealed a general better SNR for the 20-channel head coil (Fig 2A, marked in red) over all measurements, followed by the 64-channel (in blue) and the 16-channel pediatric coil (in green). The model accordingly revealed a significant main effect of the factor coil (*F*_2,109_ = 40.75, *p* < 0.01), with a significant difference between the 16-channel pediatric coil and the 20-channel coil (*b* = 0.25, *t* = 7.11, *p* < 0.01), but no difference between the 16-channel pediatric coil and the 64-channel coil (*b* = -0.04, *t* = 1.25, *p* = 0.21). Notably, when the prescan normalize filter was ON, SNR differences across coils were smaller for all sequences as compared to when the prescan normalize filter was OFF. This was statistically confirmed by a model adding the two-way interaction effects between the factors sequence and prescan and coil and prescan. The two-way interaction effects between sequence and prescan was thus significant (*F*_4,103_ = 4.25, *p* < 0.01). Less differences occurred between the 64-channel and the 16-channel head coil when prescan normalize filter was ON, which was statistically significant as indicated by the two-way interaction between the coil and prescan (*F*_2,103_ = 15.88, *p* < 0.01; see also Fig 2A).

The usage of the multiband sequences (CMRR3, CMRR8 and MB3) compared to standard singleband sequence (EPI) did not result in any difference regarding the SNR. However, it needs to be considered that more data points can be acquired within the same time, since multiband reduces the TR and consequently the acquisition time (see Table 2).

**Fig 3:**
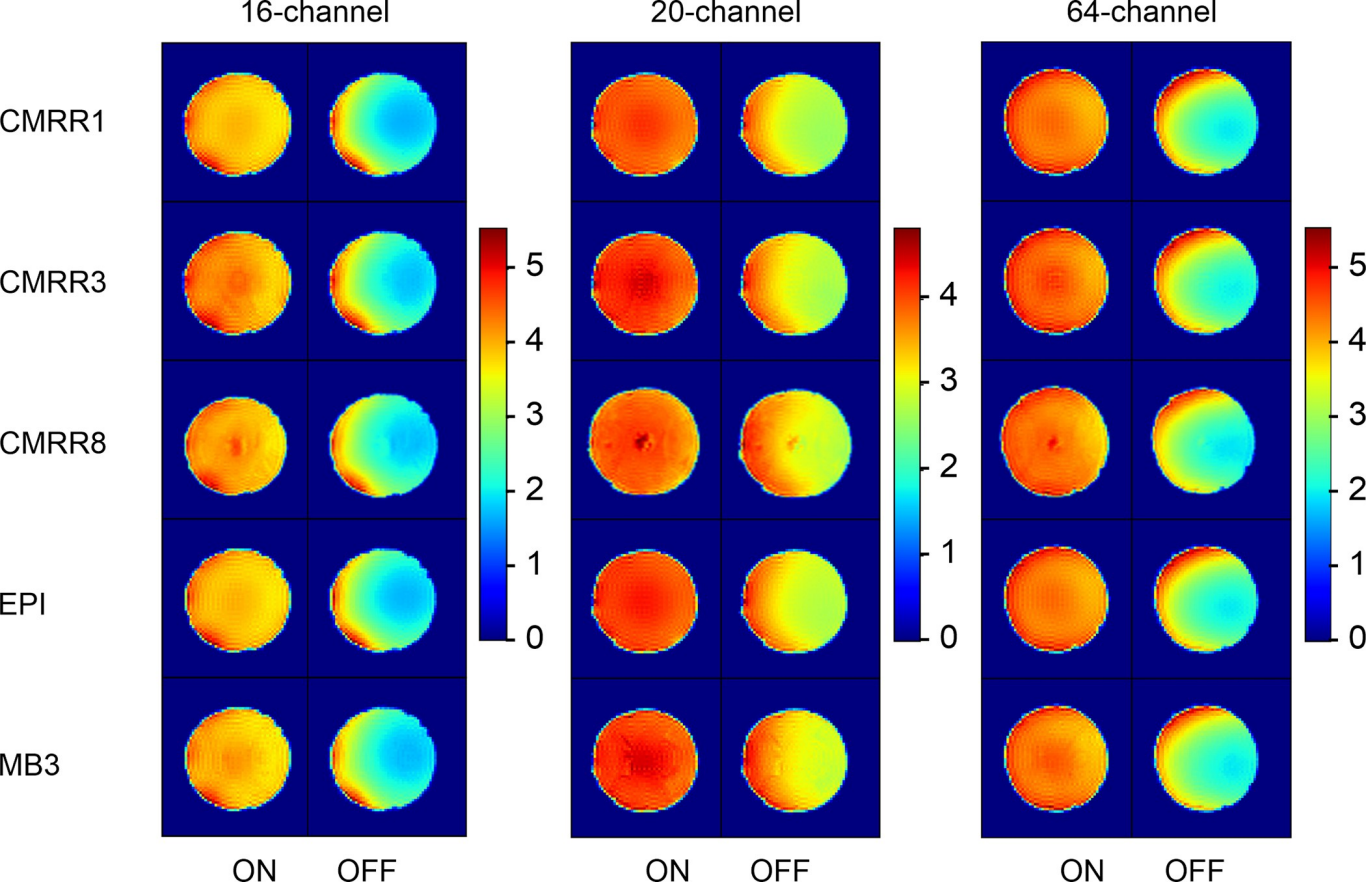
SNR homogeneity maps shown for the middle slice of the phantom for the different sequences (in rows) and head coils (in columns), with separation of the prescan normalize filter (ON in each left column and OFF in each right column).

Homogeneity maps of the SNR (Fig 3) for each measurement and head coil indicated that the images were more homogenous when the prescan normalize filter was used.

### tSNR

In contrast to the SNR calculation, where the higher SNR values were found for the 20-channel head coil, we found better tSNR values for the 16-channel head coil followed by the 20- and the 64-channel ones (Fig 2B and S1 Table). Accordingly, the model with tSNR as the dependent variable showed a significant difference between the 16- and 20-channel head coil (*b* = -36.50, *t* = 11.04, *p* < 0.01) and between the 16- and 64-channel head coil (*b* = -31.47, *t* = 9.52, *p* < 0.01). Further, there are no substantial differences between prescan normalize ON and OFF throughout the sequences (*F*_1,109_ = 0.22, *p* = 0.64), but larger tSNR occurred for the standard EPI and CMRR1 compared to the multiband sequences (*F*_4,109_ = 577.52, *p* < 0.01), with about twofold higher values.

**Fig 4:**
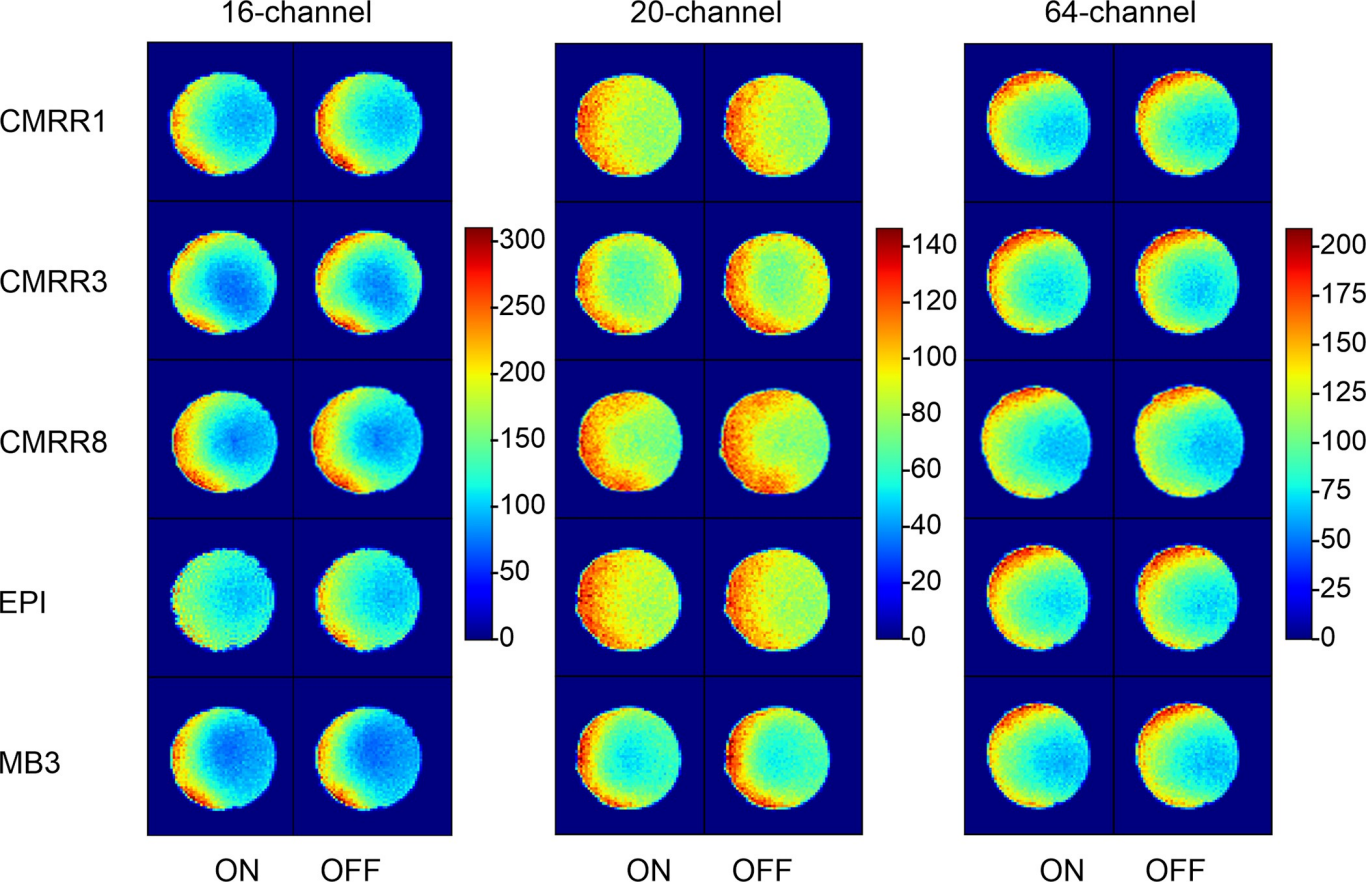
tSNR homogeneity maps shown for the middle slice of the phantom for the different sequences (in rows) and head coils (in columns), with separation of the prescan normalize filter (ON in each left column and OFF in each right column).

Homogeneity maps of the tSNR (Fig 4) for each measurement and head coil indicated differences in the homogeneities between the different head coils with more homogeneous results for the 20-channel head coils and clear differences between the singleband versus multiband sequences in the 16-channel head coil.

## Discussion

Over the past few decades, our understanding of the structure and function of the brain in newborns and very young children has increased dramatically. fMRI of the newborn human brain is currently employed in clinical and research settings for a widespread of diagnostic and scientific purposes. BOLD changes following neuronal activation only account for a small proportion of changes in raw fMRI signal intensity. The remaining variance in fMRI signal intensity is due to a widespread of factors, in general referred to as noise. Establishing practices to cope with such noise and other difficulties of neonatal fMRI constitutes a fruitful path to enrich our understanding of the developing brain and the human brain in general. Given the lack of clear recommendations on sequences, protocol parameters and head coils for neonatal fMRI, the current study compared the SNR and tSNR of different sequences and protocol parameters in combination with three different head coils, the 20- and 64-channel adult head coil and the 16-channel pediatric head coil. In line with the neonatal and general MRI literature, we hypothesized that the application of a prescan normalize filter, and the use of standard (non-multiband) sequences and smaller head coils would result in better SNR and tSNR measures.

Regarding SNR, our findings are partly in line with prior expectations. Overall, the usage of the prescan normalize filter entails an advantage in terms of SNR. As already shown in Schmitt and Rieger [33], this is likely due to a complex-values multiplication of smoothed images instead of a mere scalar multiplication of image intensities. The number of elements in the head coil, as well as the filling factor was not linearly related to SNR. The 20-channel head coil showing some advantages in all conditions over the 64-channel and, unexpectedly, over the dedicated pediatric head. This conjecture has relevant practical implications for research centers where no funds for additional MRI equipment are available and only adult coils are at disposal. One possible explanation is related to the difference in the number of channels in each coil. According to the literature, increasing number of channels as well as a higher filling factor should improve the SNR which should have result in higher SNR values for the 64-channel head coil, because of the higher number of elements and a better filling factor. Ghotra et al. [46] build a size-adaptive 32-channel array coil for infants which resulted in twofold higher SNR values compared to a commercially available 32-channel head coil, because of the better filling factor. Nevertheless, Kaza [47] and Schmitt and Rieger [33] also found that SNR might differ depending on the region in the brain, i.e. head coils with more channels are less sensitive in subcortical regions. Consequently, the fact that only a small phantom is used, probably mimics the fact that less SNR is found in subcortical regions in head coils with a larger number of elements.

Another potential explanation for the fact, that the performance of the pediatric head coil is not as expected, is the geometry of the phantom, as it is not a perfect match of a newborn head’s shape as well as the tissue probabilities might not be the same as in “real” brains, since the phantom only contains one specific type of filling, not allowing for any contrast within the image. In addition, in adult head coils, normally the shoulders of the adults touch the lower end of the coil surface. For newborns it might not be possible to position the head exactly in the middle of the coil as it was done with the phantom, because the shoulders might not fit that well. A logical better alternative is to test the performance with human neonates, however, as mentioned before it is rarely possible to conduct pilot studies with newborns or infants, hence the need of using phantoms for sequence adjustment. It might thus remain necessary to adjust some of the parameters during the study.

With respect to tSNR, our findings largely support our expectation that the pediatric head coil would outperform the others. This was the case irrespective of the application of a prescan normalize filter and more prominent in non-multiband (“CMRR1” and standard “EPI”) sequences and in line with adult studies showing that head coils with less channels improve data quality in functional scans [33]. Finally, in line with prior expectations and other studies [31], multiband sequences shower poorer tSNR, altogether, we observed a considerable advantage in tSNR when using a pediatric head coil.

Besides the number of the channels and the geometry of the phantom and the arrangement within the coil, there are also various parameters in the protocol which might explain the differences in the SNR and tSNR, such as bandwidth, flip angle and size of the acquisition matrix as well as resolution. As shown in Table 2, the resolution and the voxel size of most of the sequences used in the current study was the same, i.e. 2.5 mm^3^. The only difference is the CMRR8 protocol which was originally taken from the HCP. There, the voxel size is a bit smaller with 2 mm^3^ and thus, the resolution larger. Smaller voxel sized lead to a decrease in the signal because there are fewer protons within each voxel, as seen in the tSNR results where the values for the CMRR8 sequence are the lowest. Another factor which might influence the SNR and tSNR is the bandwidth, where a higher bandwidth led to an increased amount of noise captured within the signal. Tn the current study, the bandwidth does not influence the SNR or tSNR in a direct way, as it was similar for EPI, CMRR1 and CMRR3 with 1700 Hz compared to the MB3 with 2790 Hz. But there was no difference in the data between those sequences. Finally, also the flip angle varies between the protocols, however still depending on the TR to allow for the optimal flip angle (Ernst angle) to maximize the signal by balancing transverse magnetization and longitudinal recovery. Thus, setting the flip angle at the optimal level, it should not influence the SNR or tSNR calculation.

### General recommendations

Based on the results conveyed in this study, we propose the following guidelines to assist researchers in the early stages of designing a research project on newborns. We hope that they prove useful, in particular when there is no prior experience with neonatal MRI in the lab and as it is generally the case, optimizing MRI sequences with human neonates is not feasible.

1. Whenever possible, we recommend considering the use of a dedicated pediatric head coil, because of the more appropriate filling factor and resulting better tSNR. However, if this is not an option, adult head coils may be employed too, considering that SNR values do not differ dramatically between various head coils.
2. Whenever possible, imaging protocols should include a prescan normalize filter, as this has been shown to result in higher SNR.
3. The standard EPI imaging protocol shows a better performance in tSNR compared to a multiband sequence. However, in the common scenario of very limited scan time, fast rs-fMRI acquisition in neonates using the multiband sequence protocol may outweigh the tSNR advantage of EPI sequences. Specifically, the multiband sequence takes less than half the time to acquire the same amount of data (i.e., images).
4. Whenever possible, phantom studies should be considered to develop an optimized environment for neonatal fMRI studies.

### Limitations

The present study assessed SNR and tSNR from various MRI sequences proposed in the scientific literature and using three different head coils with the aim of providing practical guidelines for MRI users when planning imaging studies on newborns. One important limitation of the study however is that the observations and conclusions derived from the present work are mostly applicable to scenarios that closely mimic the present methodology (i.e., same MRI scanner vendor, MRI sequences, and head coils). Additionally, there are currently no specific protocols for the pediatric head coil, especially for fMRI. Furthermore, it is difficult to adjust protocol parameters on a phantom, since not every important factor could be considered, as for example, the contrast-to-noise ratio, which cannot be calculated in a homogenous phantom. Nevertheless, unlike studies in adults, where it is not as ethically problematic to perform a pilot measurement and adjust some protocol parameters, this is not possible in neonates or infants for reasons of protection. Thus, recommendations need to be derived from measurements with phantoms. This study fills an important gap in the lack of a similar published guideline for the field of newborn fMRI.

## Supporting information

S1 Table Absolute values for the A) SNR and B) tSNR for the different sequences and coil combinations in 4 different runs, with separation for prescan normalize filter ON and OFF.
(DOCX)

## References

[1] Dubois J, Alison M, Counsell SJ, Hertz‐Pannier L, Hüppi PS, Benders MJ. MRI of the Neonatal Brain. A review of methodological challenges and neuroscientific advances. J Magn Reson Imaging. 2021; 53:1318–43. doi: 10.1002/jmri.27192 32420684 PMC8247362

[2] Feng K, Rowell AC, Andres A, Bellando BJ, Lou X, Glasier CM, et al. Diffusion tensor MRI of white matter of healthy full-term newborns. Relationship to neurodevelopmental outcomes. Radiology. 2019; 292:179–87. Epub 2019/06/04. doi: 10.1148/radiol.2019182564 . 31161971 PMC6614910

[3] Lebel C, Deoni S. The development of brain white matter microstructure. NeuroImage. 2018; 182:207–18. Epub 2018/01/03. doi: 10.1016/j.neuroimage.2017.12.097 . 29305910 PMC6030512

[4] Qiu A, Mori S, Miller MI. Diffusion tensor imaging for understanding brain development in early life. Annual Review of Psychology. 2015; 66:853–76. doi: 10.1146/annurev-psych-010814-015340 . 25559117 PMC4474038

[5] Zhao T, Xu Y, He Y. Graph theoretical modeling of baby brain networks. NeuroImage. 2019; 185:711–27. doi: 10.1016/j.neuroimage.2018.06.038 . 29906633

[6] Arichi T. Functional MRI of the developing neonatal brain. Potential and challenges for the future. Developmental Medicine & Child Neurology. 2012; 54:680. doi: 10.1111/j.1469-8749.2012.04355.x 22715977

[7] Cai Y, Wu X, Su Z, Shi Y, Gao J-H. Functional thalamocortical connectivity development and alterations in preterm infants during the neonatal period. Neuroscience. 2017; 356:22–34. doi: 10.1016/j.neuroscience.2017.05.011 28526574

[8] Lubsen J, Vohr B, Myers E, Hampson M, Lacadie C, Schneider KC, et al. Microstructural and functional connectivity in the developing preterm brain. Seminars in Perinatology. 2011; 35:34–43. doi: 10.1053/j.semperi.2010.10.006 . 21255705 PMC3063450

[9] Zhang H, Shen D, Lin W. Resting-state functional MRI studies on infant brains. A decade of gap-filling efforts. NeuroImage. 2019; 185:664–84. doi: 10.1016/j.neuroimage.2018.07.004 29990581 PMC6289773

[10] Heep A, Scheef L, Jankowski J, Born M, Zimmermann N, Sival D, et al. Functional magnetic resonance imaging of the sensorimotor system in preterm infants. Pediatrics. 2009; 123:294–300. doi: 10.1542/peds.2007-3475 . 19117895

[11] Scheef L, Nordmeyer-Massner JA, Smith-Collins AP, Müller N, Stegmann-Woessner G, Jankowski J, et al. Functional laterality of task-evoked activation in sensorimotor cortex of preterm infants. An optimized 3 T fMRI study employing a customized neonatal head coil. PLoS ONE. 2017; 12:e0169392. doi: 10.1371/journal.pone.0169392 . 28076368 PMC5226735

[12] Merhar SL, Gozdas E, Tkach JA, Harpster KL, Schwartz TL, Yuan W, et al. Functional and structural connectivity of the visual system in infants with perinatal brain injury. Pediatric Research. 2016; 80:43–8. doi: 10.1038/pr.2016.49 26991261

[13] Erberich SG, Friedlich P, Seri I, Nelson MD, Blüml S. Functional MRI in neonates using neonatal head coil and MR compatible incubator. NeuroImage. 2003; 20:683–92. doi: 10.1016/S1053-8119(03)00370-7 14568444

[14] Fitzgibbon SP, Harrison SJ, Jenkinson M, Baxter L, Robinson EC, Bastiani M, et al. The developing Human Connectome Project (dHCP) automated resting-state functional processing framework for newborn infants. NeuroImage. 2020; 223:117303. Epub 2020/08/29. doi: 10.1016/j.neuroimage.2020.117303 . 32866666 PMC7762845

[15] Arthurs OJ, Edwards A, Austin T, Graves MJ, Lomas DJ. The challenges of neonatal magnetic resonance imaging. Pediatric Radiology. 2012; 42:1183–94. doi: 10.1007/s00247-012-2430-2 22886375

[16] Dagia C, Ditchfield M. 3T MRI in paediatrics. Challenges and clinical applications. European Journal of Radiology. 2008; 68:309–19. doi: 10.1016/j.ejrad.2008.05.019 18768276

[17] Enguix V, Ding Y, Lodygensky GA. Recent advances in preclinical and clinical multimodal MR in the newborn brain. Journal of Magnetic Resonance. 2018; 292:149–54. doi: 10.1016/j.jmr.2018.04.017 29731237

[18] Hillenbrand CM, Reykowski A. MR imaging of the newborn. A technical perspective. Magnetic Resonance Imaging Clinics of North America. 2012; 20:63–79. doi: 10.1016/j.mric.2011.10.002 . 22118593

[19] Srinivasan L, Rutherford MA. MRI of the newborn brain. Paediatrics and Child Health. 2008; 18:183–95. doi: 10.1016/j.paed.2008.01.003

[20] Tocchio S, Kline-Fath B, Kanal E, Schmithorst VJ, Panigrahy A. MRI evaluation and safety in the developing brain. Seminars in Perinatology. 2015; 39:73–104. doi: 10.1053/j.semperi.2015.01.002 25743582 PMC4380813

[21] Xu Y, Morel B, Dahdouh S, Puybareau É, Virzì A, Urien H, et al. The challenge of cerebral magnetic resonance imaging in neonates. A new method using mathematical morphology for the segmentation of structures including diffuse excessive high signal intensities. Medical Image Analysis. 2018; 48:75–94. doi: 10.1016/j.media.2018.05.003 29852312

[22] Makropoulos A, Aljabar P, Wright R, Hüning B, Merchant N, Arichi T, et al. Regional growth and atlasing of the developing human brain. NeuroImage. 2016; 125:456–78. doi: 10.1016/j.neuroimage.2015.10.047 26499811 PMC4692521

[23] Orasanu E, Melbourne A, Cardoso MJ, Modat M, Taylor AM, Thayyil S, et al. Brain volume estimation from post-mortem newborn and fetal MRI. NeuroImage: Clinical. 2014; 6:438–44. doi: 10.1016/j.nicl.2014.10.007 25379457 PMC4218943

[24] Dubois J, Dehaene-Lambertz G, Kulikova S, Poupon C, Hüppi PS, Hertz-Pannier L. The early development of brain white matter. A review of imaging studies in fetuses, newborns and infants. Neuroscience. 2014; 276:48–71. Epub 2013/12/28. doi: 10.1016/j.neuroscience.2013.12.044 . 24378955

[25] Hughes EJ, Winchman T, Padormo F, Teixeira R, Wurie J, Sharma M, et al. A dedicated neonatal brain imaging system. Magnetic Resonance in Medicine. 2017; 78:794–804. doi: 10.1002/mrm.26462 27643791 PMC5516134

[26] Patkee PA, Baburamani AA, Kyriakopoulou V, Davidson A, Avini E, Dimitrova R, et al. Early alterations in cortical and cerebellar regional brain growth in Down Syndrome. An in vivo fetal and neonatal MRI assessment. NeuroImage: Clinical. 2020; 25:102139. doi: 10.1016/j.nicl.2019.102139 31887718 PMC6938981

[27] Corea J, Ye P, Seo D, Butts-Pauly K, Arias AC, Lustig M. Printed receive coils with high Acoustic transparency for magnetic resonance guided focused ultrasound. Scientific Reports. 2018; 8:3392. doi: 10.1038/s41598-018-21687-1 29467432 PMC5821831

[28] Xie J, You X, Huang Y, Ni Z, Wang X, Li X, et al. 3D-printed integrative probeheads for magnetic resonance. Nature Communications. 2020; 11:5793. doi: 10.1038/s41467-020-19711-y 33188186 PMC7666178

[29] Zamarayeva AM, Gopalan K, Corea JR, Liu MZ, Pang K, Lustig M, et al. Custom, spray coated receive coils for magnetic resonance imaging. Scientific Reports. 2021; 11:2635. doi: 10.1038/s41598-021-81833-0 33514816 PMC7846777

[30] Smith-Collins APR, Luyt K, Heep A, Kauppinen RA. High frequency functional brain networks in neonates revealed by rapid acquisition resting state fMRI. Human Brain Mapping. 2015; 36:2483–94. doi: 10.1002/hbm.22786 . 25787931 PMC6869609

[31] Seidel P, Levine SM, Tahedl M, Schwarzbach JV. Temporal signal-to-noise changes in combined multislice- and in-plane-accelerated echo-planar imaging with a 20- and 64-channel coil. Scientific Reports. 2020; 10:5536. doi: 10.1038/s41598-020-62590-y 32218476 PMC7099092

[32] Montin E, Lattanzi R. Seeking a Widely Adoptable Practical Standard to Estimate Signal-to-Noise Ratio in Magnetic Resonance Imaging for Multiple-Coil Reconstructions. J Magn Reson Imaging. 2021; 54:1952–64. Epub 2021/07/04. doi: 10.1002/jmri.27816 . 34219312 PMC8633048

[33] Schmitt T, Rieger JW. Recommendations of choice of head coil and prescan normalize filter depend on region of interest and task. Frontiers in Neuroscience. 2021; 15:735290. doi: 10.3389/fnins.2021.735290 34776844 PMC8585748

[34] Welvaert M, Rosseel Y. On the definition of signal-to-noise ratio and contrast-to-noise ratio for fMRI data. PLoS ONE. 2013; 8:e77089. doi: 10.1371/journal.pone.0077089 . 24223118 PMC3819355

[35] Dietrich O, Raya JG, Reeder SB, Ingrisch M, Reiser MF, Schoenberg SO. Influence of multichannel combination, parallel imaging and other reconstruction techniques on MRI noise characteristics. Magnetic Resonance Imaging. 2008; 26:754–62. Epub 2008/04/28. doi: 10.1016/j.mri.2008.02.001 . 18440746

[36] Dietrich O, Raya JG, Reeder SB, Reiser MF, Schoenberg SO. Measurement of signal-to-noise ratios in MR images: influence of multichannel coils, parallel imaging, and reconstruction filters. J Magn Reson Imaging. 2007; 26:375–85. doi: 10.1002/jmri.20969 . 17622966

[37] Parrish TB, Gitelman DR, LaBar KS, Mesulam M-M. Impact of signal-to-noise on functional MRI. Magnetic Resonance in Medicine. 2000; 44:925–32. doi: 10.1002/1522-2594(200012)44:6&lt;925::aid-mrm14&gt;3.0.co;2-m 11108630

[38] Wiggins GC, Triantafyllou C, Potthast A, Reykowski A, Nittka M, Wald LL. 32-channel 3 Tesla receive-only phased-array head coil with soccer-ball element geometry. Magnetic Resonance in Medicine. 2006; 56:216–23. doi: 10.1002/mrm.20925 . 16767762

[39] Howell BR, Styner MA, Gao W, Yap P-T, Wang L, Baluyot K, et al. The UNC/UMN Baby Connectome Project (BCP). NeuroImage. 2019; 185:891–905. Epub 2018/03/22. doi: 10.1016/j.neuroimage.2018.03.049 . 29578031 PMC6151176

[40] Feinberg DA, Moeller S, Smith SM, Auerbach E, Ramanna S, Glasser MF, et al. Multiplexed echo planar imaging for sub-Second whole brain fMRI and fast diffusion imaging. PLoS ONE. 2010; 5:e15710. doi: 10.1371/journal.pone.0015710 21187930 PMC3004955

[41] Xu J, Moeller S, Auerbach EJ, Strupp J, Smith SM, Feinberg DA, et al. Evaluation of slice accelerations using multiband echo planar imaging at 3T. NeuroImage. 2013; 83:991–1001. doi: 10.1016/j.neuroimage.2013.07.055 23899722 PMC3815955

[42] Breuer FA, Kannengiesser SAR, Blaimer M, Seiberlich N, Jakob PM, Griswold MA. General formulation for quantitative G-factor calculation in GRAPPA reconstructions. Magnetic Resonance in Medicine. 2009; 62:739–46. doi: 10.1002/mrm.22066 . 19585608

[43] Talagala SL, Sarlls JE, Liu S, Inati SJ. Improvement of temporal signal-to-noise ratio of GRAPPA accelerated echo planar imaging using a FLASH based calibration scan. Magnetic Resonance in Medicine. 2016; 75:2362–71. Epub 2015/07/20. doi: 10.1002/mrm.25846 . 26192822 PMC4720593

[44] Duyn JH. The future of ultra-high field MRI and fMRI for study of the human brain. NeuroImage. 2012; 62:1241–8. doi: 10.1016/j.neuroimage.2011.10.065 22063093 PMC3389184

[45] Esteban O, Birman D, Schaer M, Koyejo OO, Poldrack RA, Gorgolewski KJ. MRIQC. Advancing the automatic prediction of image quality in MRI from unseen sites. PLoS ONE. 2017; 12. doi: 10.1371/journal PMC5612458 28945803

[46] Ghotra A, Kosakowski HL, Takahashi A, Etzel R, May MW, Scholz A, et al. A size-adaptive 32-channel array coil for awake infant neuroimaging at 3 Tesla MRI. Magnetic Resonance in Medicine. 2021; 86:1773–85. Epub 2021/04/08. doi: 10.1002/mrm.28791 . 33829546

[47] Kaza E, Klose U, Lotze M. Comparison of a 32-channel with a 12-channel head coil: are there relevant improvements for functional imaging. J Magn Reson Imaging. 2011; 34:173–83. Epub 2011/05/25. doi: 10.1002/jmri.22614 . 21618334

