# Supplementary material for: Customization of neonatal functional magnetic resonance imaging: A preclinical phantom-based study
Source: PLoS One. 2024 Nov 1;19(11):e0313192. doi: 10.1371/journal.pone.0313192 (PMC11530025; doi:10.1371/journal.pone.0313192)
Supplement: S1 Table — Absolute values for the A) SNR and B) tSNR for the different sequences and coil combinations in 4 different runs, with separation for prescan normalize filter ON and OFF. (DOCX) [file pone.0313192.s001.docx]

1. SNR

|  |  | 16-channel | | 20-channel | | 64-channel | |
| --- | --- | --- | --- | --- | --- | --- | --- |
|  |  | ON | OFF | ON | OFF | ON | OFF |
| CMRR1 | 1 | 3.36 | 2.14 | 3.61 | 2.91 | 3.41 | 2.51 |
|  | 2 | 3.27 | 2.21 | 3.42 | 2.35 | 3.28 | 1.73 |
|  | 3 | 3.30 | 2.19 | 3.46 | 2.48 | 3.31 | 2.03 |
|  | 4 | 3.29 | 2.09 | 3.37 | 2.30 | 3.33 | 1.99 |
| CMRR3 | 1 | 3.34 | 2.13 | 3.56 | 2.91 | 3.37 | 2.49 |
|  | 2 | 3.22 | 2.17 | 3.34 | 2.30 | 3.21 | 1.70 |
|  | 3 | 3.25 | 2.13 | 3.41 | 2.44 | 3.26 | 1.99 |
|  | 4 | 3.25 | 2.05 | 3.32 | 2.19 | 3.28 | 1.95 |
| CMRR8 | 1 | 3.69 | 2.24 | 3.93 | 3.16 | 3.80 | 2.65 |
|  | 2 | 3.63 | 2.27 | 3.73 | 2.43 | 3.53 | 1.74 |
|  | 3 | 3.65 | 2.26 | 3.81 | 2.61 | 3.64 | 2.07 |
|  | 4 | 3.59 | 2.15 | 3.70 | 2.29 | 3.64 | 2.03 |
| EPI | 1 | 3.40 | 2.17 | 3.62 | 2.96 | 3.42 | 2.52 |
|  | 2 | 3.32 | 2.22 | 3.42 | 2.37 | 3.29 | 1.74 |
|  | 3 | 3.32 | 2.19 | 3.45 | 2.50 | 3.35 | 2.04 |
|  | 4 | 3.35 | 2.11 | 3.39 | 2.31 | 3.34 | 2.01 |
| MB3 | 1 | 3.39 | 2.15 | 3.61 | 2.96 | 3.39 | 2.52 |
|  | 2 | 3.27 | 2.20 | 3.37 | 2.33 | 3.23 | 1.71 |
|  | 3 | 3.34 | 2.17 | 3.42 | 2.48 | 3.31 | 2.01 |
|  | 4 | 3.29 | 2.08 | 3.34 | 2.22 | 3.28 | 1.98 |

1. tSNR

|  |  | 16-channel | | 20-channel | | 64-channel | |
| --- | --- | --- | --- | --- | --- | --- | --- |
|  |  | ON | OFF | ON | OFF | ON | OFF |
| CMRR1 | 1 | 264.39 | 261.90 | 202.46 | 199.54 | 183.17 | 181.10 |
|  | 2 | 288.66 | 289.19 | 238.20 | 235.38 | 242.69 | 239.98 |
|  | 3 | 285.51 | 287.46 | 230.87 | 228.32 | 209.28 | 207.23 |
|  | 4 | 291.40 | 289.29 | 251.42 | 243.35 | 213.94 | 211.18 |
| CMRR3 | 1 | 124.58 | 122.47 | 105.76 | 104.84 | 121.22 | 119.91 |
|  | 2 | 164.39 | 164.30 | 113.38 | 112.30 | 151.29 | 148.71 |
|  | 3 | 175.31 | 172.97 | 114.88 | 113.92 | 137.77 | 136.16 |
|  | 4 | 165.19 | 162.34 | 128.84 | 127.31 | 139.23 | 137.42 |
| CMRR8 | 1 | 73.70 | 72.27 | 51.55 | 50.95 | 58.12 | 57.24 |
|  | 2 | 92.53 | 90.63 | 56.05 | 55.28 | 82.80 | 81.07 |
|  | 3 | 97.74 | 95.40 | 55.72 | 54.94 | 70.41 | 69.09 |
|  | 4 | 90.67 | 88.64 | 70.49 | 69.32 | 71.51 | 70.23 |
| EPI | 1 | 214.51 | 234.66 | 202.82 | 200.11 | 179.11 | 178.67 |
|  | 2 | 262.13 | 275.23 | 220.17 | 231.81 | 246.27 | 242.42 |
|  | 3 | 205.45 | 248.21 | 225.46 | 227.57 | 196.88 | 202.74 |
|  | 4 | 191.53 | 236.30 | 214.58 | 233.26 | 209.94 | 210.13 |
| MB3 | 1 | 109.37 | 107.85 | 76.07 | 75.13 | 88.90 | 87.85 |
|  | 2 | 138.60 | 138.15 | 87.53 | 86.44 | 126.86 | 125.19 |
|  | 3 | 142.95 | 141.59 | 84.50 | 83.52 | 108.10 | 107.21 |
|  | 4 | 136.08 | 135.15 | 102.97 | 101.73 | 110.19 | 108.87 |

Table S1: Absolute values for the A) SNR and B) tSNR for the different sequences and coil combinations in 4 different runs, with separation for prescan normalize filter ON and OFF.
